# Racial differences and geographic variations in oral anticoagulation treatment among Medicare patients with non-valvular atrial fibrillation

**DOI:** 10.1371/journal.pone.0314345

**Published:** 2024-12-12

**Authors:** Larry R. Jackson, Amiee Kang, Virginia Noxon, Nipun Atreja, Dionne M. Hines, Melissa Hagan, Jenny Jiang, Brett D. Atwater

**Affiliations:** 1 Duke Clinical Research Institute, Durham, NC, United States of America; 2 Bristol Myers Squibb, Lawrenceville, NJ, United States of America; 3 STATinMED, LLC, Dallas, TX, United States of America; 4 Pfizer, New York, NY, United States of America; 5 Inova Schar Heart and Vascular, Falls Church, VA, United States of America; Sapienza University of Rome: Universita degli Studi di Roma La Sapienza, ITALY

## Abstract

**Introduction:**

Use of oral anticoagulants (OACs) for stroke reduction in atrial fibrillation (AF) varies by race and geography within the United States. We seek to better understand the relationship between OAC underutilization, race, and US geography.

**Methods:**

Patients with AF were selected from the US Centers for Medicare & Medicaid Services claims database from January 1, 2013, to December 31, 2016. The final population consisted of patients with 12 months of health plan enrollment before and after their index AF diagnosis, with a baseline CHAD_2_S_2_-VASc ≥2 and of either Black or White race (other races are underrepresented in the data). Among those with AF that met the inclusion criteria, patients who were prescribed warfarin or DOACs within 12 months after the index date were extracted. Each patient was assigned to a US county based on their 5-digit zip code and OAC use was stratified by race. Statistically significant differences were determined by student’s t-test and chi-square.

**Results:**

Of the 2,390,830 final patients, 94.1% were White and 5.9% were Black patients. Mean (SD) age and HASBLED scores were 78 (9) and 3.9 (1.2) respectively, for Black patients and 80 (9) and 3.3 (1.2), respectively, for White patients (p<0.0001). The mean (SD) CHAD_2_S_2_-VASc scores were 4.5 (1.9) for White patients, and 5.3 (1.9) for Black patients with p<0.0001, respectively. Black patients (vs White patients) had a higher non-treatment (no DOAC or warfarin) rate (56.1% vs 47.4%, p<0.0001) across the US which was particularly notable in the southeast. In addition, treatment rates were highly variable within each US state. Counties with dense population more frequently demonstrated significant differences by race than counties with sparse population.

**Conclusion:**

Our study showed differences in the use of OACs across US counties and among various racial groups. These disparities highlighted the areas of unmet need for both Black and White patients.

## Introduction

By 2030, AF is projected to affect 12.1 million people in the United States (US) [1]. Mortality and morbidity rates are substantial due to the incidence of ischemic stroke among patients with AF, which is approximately 5 times higher than in the general population [2, 3]. AF is responsible for 20–25% of the ischemic stroke burden in the US and is a potent driver of cardiac dysrhythmia hospitalizations in the southeast US [4]. The incidence of ischemic stroke varies by US region with the highest incidence in the southeast which contains states in the “stroke belt” [5–8]. A study of Medicare patients residing in the “stroke belt” showed that AF increases the cost of stroke hospitalizations by at least 9.4% [9]. Patients residing in these stroke belt states (Alabama, Arkansas, Georgia, Indiana, Kentucky, Louisiana, Mississippi, North Carolina, South Carolina, Tennessee, and Virginia) had a 27% higher age-adjusted stroke mortality versus those residing outside this region [10, 11]. The high incidence and mortality rates of stroke in the southeastern US coincide with the highest disability-adjusted life years (DALYs) for overall stroke and ischemic stroke in the US [5]. As the prevalence of AF increases, accelerated efforts are needed to mitigate the burden of ischemic stroke through risk factor control and increased utilization of stroke reduction therapies for patients with AF.

While oral anticoagulants (OACs) are recommended for patients with AF and increased stroke risk, OACs remain under-utilized in the US. OAC utilization varies across studies but ranges from 50%-60% of treatment-eligible patients with AF who have a moderate or high risk of ischemic stroke/systemic embolism (SE) [12–14]. Lower rates of use are noted among Black patients [10, 11, 15, 16] and those residing in the southeastern US [15].

An improved understanding of the intersection of racial and geographic variation in the use of OACs may provide opportunities to improve OAC utilization. Racial and ethnic minoritized groups with AF have been reported to have higher incidence of ischemic stroke coupled with a decreased likelihood of receiving an OAC [16–19]. The lack of detail provided in prior studies of geographic variation in OAC use and paucity of information on the relationship between geographic variation and race impedes stakeholders from identifying patient groups in specific regions that would benefit most from quality improvement strategies aimed at increasing OAC use in patients with AF [15]. While the current studies have often focused on a single factor, such as race or geographic location, our retrospective analysis was conducted to describe racial differences and geographic variation using county-level claims data in OAC treatment use among Medicare patients with AF.

## Methods

### Study design and data source

This real-world retrospective analysis utilized the US Centers for Medicare & Medicaid Services (CMS) database for the period January 1, 2012—December 31, 2017. The identification period was from January 1, 2013, to December 31, 2016. This allowed us to evaluate baseline characteristics and outcomes in the follow up period. The data included hospital inpatient, outpatient, Medicare carrier, Part D pharmacy, skilled nursing facility, home health agency, and durable medical equipment files containing medical and pharmacy claims from the 100% national Medicare fee-for-service population. The zip codes of the patients’ primary place of residence at the 5-digit level were converted to the county level. Only those with valid county information (5-digit zip code corresponding with county number) were included in the final analysis. International Classification of Diseases, 9/10^th^ Revision, Clinical Modification (ICD-9/10-CM) diagnosis and procedure codes (ICD-10-PCS), Health Care Common Procedure Coding System (HCPCS), and Current Procedural Terminology (CPT) codes were used to assess medical claims, and National Drug Codes (NDC) were used to identify prescription drug utilization.

This observational study was conducted under the provisions of Privacy Rule 45 CFR 164.514(e). The study was exempt from institutional review board review and approval since there was no collection or use of personally identifiable information in the conduct of this study. Fully anonymized data were accessed 10 October 2019 for research purposes and authors had no access to information that could identify individual participants during or after data collection.

### Study population

Patients were identified as a subset from prior analyses that observed geographic variation and temporal trends in OAC treatment among Medicare patients with AF [20, 21]. Patients with at least one inpatient or two outpatient medical claims with an AF diagnosis in any position between 7 and 365 days apart, from January 1, 2013, through December 31, 2016 (identification period) were selected for analysis. The index date was date of the first AF claim during the identification period. Patients were included for analysis if they were ≥65 years of age on the index date and had 12 months of continuous health plan enrollment with medical and pharmacy benefits (Parts A, B, and D) pre- (baseline period) and post-index date (follow-up period, including index date). Patients with rheumatic mitral valvular heart disease or valve replacement procedures (identified by ICD codes) during the baseline period were excluded in order to assess non-valvular AF (NVAF). Patients were followed from the index date until at least 12 months after the index date, Medicare disenrollment, death, or end of study period. Patients who died during the 12-month follow-up period were included in the study sample (11% of the initial sample) to avoid selection bias. In addition, only patients with a valid county, a CHA_2_DS_2_-VASc score ≥2 in the baseline period and with a race of Black or White were kept in the final sample due to small sample sizes of other races. The CHA_2_DS_2-_VASc score (scale of 0–9), which measures the risk of ischemic stroke among AF patients, was calculated based on evidence of congestive heart failure; hypertension; ≥75 years of age; diabetes mellitus; history of stroke, transient ischemic attack, or thromboembolism; vascular disease; age 65–74 years; and sex [22]. The requirement for a CHA_2_DS_2_-VASc score ≥2 was chosen based on the clinical recommendations at the time of study initiation and to reflect the guidance provided by professional societies for OAC treatment in patients with AF during data availability [23]. Prior to the update in 2019, male and female patients with AF and a CHA_2_DS_2_-VASc score ≥2 were recommended to receive OAC treatment [23, 24]. Patients who were prescribed warfarin or a direct OAC (DOAC) were identified and categorized according to the first OAC prescription filled on or after the index date.

### Study variables

County-level demographic and clinical characteristics were calculated for the 12-month baseline period. Mean Charlson Comorbidity Index (CCI), CHA_2_DS_2_-VASc, and mean modified HAS-BLED scores were reported. The modified HAS-BLED score (scale of 0–8), which estimates the 1-year risk of major bleeding for patients with AF, was calculated based on 6 characteristics: hypertension, abnormal kidney and/or liver function, stroke, bleeding, elderly (age >65), and alcohol/drug therapy. Labile international normalized ratio information, used in the standard HAS-BLED score, was not available in the data [25]. In this population, the minimum HAS-BLED and CHA_2_DS_2_-VASc scores were 1, since all patients were ≥65 years of age.

### Study outcomes

Prevalent OAC treatment use during the 12-month follow-up period (including index date), was reported separately as any OAC treatment, warfarin treatment, or DOAC (apixaban, dabigatran, rivaroxaban, and edoxaban) treatment of any dose based on the first observed OAC prescription claim (patients were assigned to OAC cohorts based on the first prescription filled on or after the index date).

### Statistical analysis

The statistical software SAS version 9.4 (SAS Institute Inc., Cary, NC, USA) was used to perform this analysis. Based on primary residence, study patients were assigned to one of the 3,143 US counties allowing them to be placed within a state based on 2017 US Census data. R mapping software was used to visualize geographic variation in OAC treatment use by race [26].

Statistically significant differences between races were determined by student’s t-test for continuous variables (age, etc.) and chi-square for categorical variables (proportion of patients in each county, etc.). Statistical significance was determined as p<0.05.

## Results

### Patient characteristics

**Fig 1:**
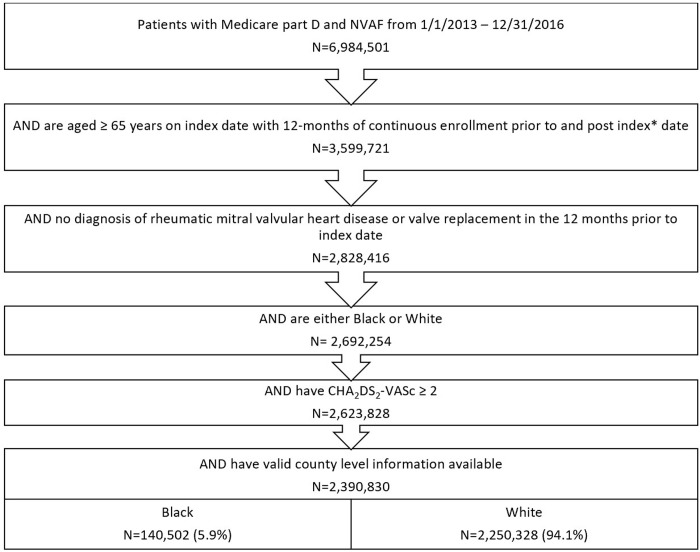
Patient selection criteria. Application of inclusion and exclusion criteria to starting patient population to show how final population was determined. Footnote: 2,895,492 patients aged 65 years or older on the index date, who did not have continuous enrollment before and after this date, were excluded.

There were 2,623,828 patients who met the initial inclusion and exclusion criteria: 94.1% were White patients while 5.9% were Black patients (**Fig 1**). Black patients were younger [mean age 78 (standard deviation (SD) 9.1)] with higher mean CCI score (4.6 [3.4]), HAS-BLED (3.9 [1.2]), and CHA_2_DS_2_-VASc (5.3 [1.9]) scores compared to White patients (mean age 80 [9] with mean scores of 3.7 [3.0], 3.3 [1.2], and 4.5 [1.9], respectively; **Table 1**).

**Table 1:** Patient characteristics.

	Black Patients		White Patients		P-Value
	Mean / N	SD/%	Mean / N	SD/%	
**All Patients**	140,502	100%	2,250,328	100%	
Age	77.6	9.1	79.5	8.6	<0.0001
**Gender**					<0.0001
Male	54,093	38.5%	996,895	44.3%	
Female	86,409	61.5%	1,253,433	55.7%	
**CHA**_**2**_ **DS**_**2**_ **-VASc Score** ^**a**^	5.3	1.9	4.5	1.9	<0.0001
**HAS-BLED Score** ^**b**^	3.9	1.2	3.3	1.2	<0.0001
**CCI Score** ^**c**^	4.6	3.4	3.7	3.0	<0.0001

^a^CHA_2_DS_2_-VASc = **C**ongestive heart failure, **H**ypertension, **A**ge ≥75, **D**iabetes, **S**troke/ transient ischemic attack, **V**ascular disease, **A**ge 65–74, **S**ex (female)

^b^HAS-BLED = **H**ypertension, **A**bnormal renal/liver function, **S**troke, **B**leeding history, **L**abile INR, **E**lderly (age >65), **D**rugs (concomitant aspirin, NSAIDs) or alcohol

^c^CCI = Charlson Comorbidity Index

### Treatment rates

**Fig 2:**
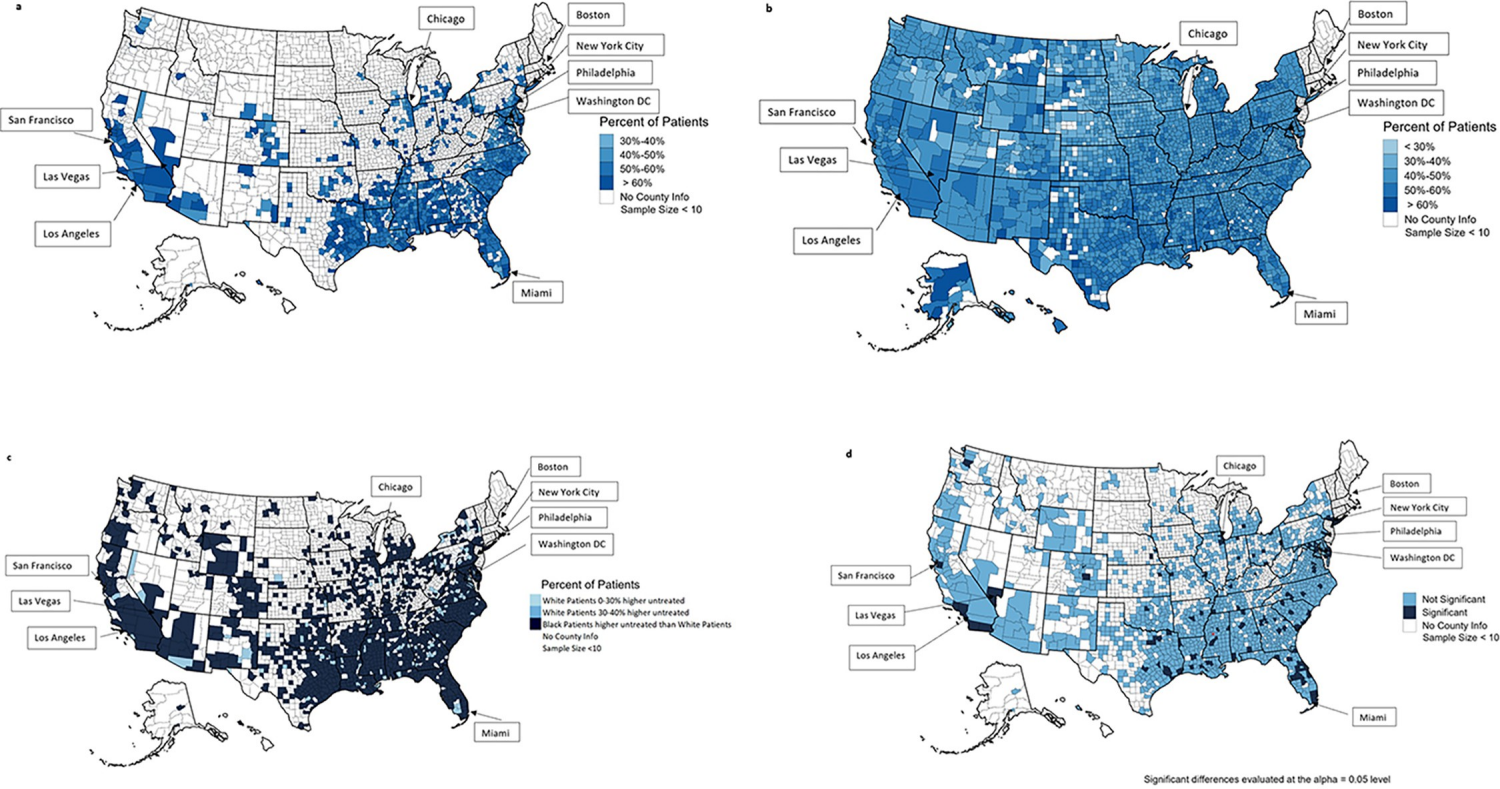
**a. Geographic variation of untreated rates among black Medicare patients with non-valvular atrial fibrillation and CHA_2_DS_2_-VASc score ≥2 (N = 140,502).** Untreated rates in each US state for Black AF subjects. CHA_2_DS_2_-VASc = **C**ongestive heart failure, **H**ypertension, **A**ge ≥75, **D**iabetes, **S**troke/ transient ischemic attack, **V**ascular disease, **A**ge 65–74, **S**ex (female). **b. Geographic variation of untreated rates among white Medicare patients with non-valvular atrial fibrillation and CHA**_**2**_**DS**_**2**_**-VASc score ≥2 (N = 2,250,328).** Untreated rates in each US state for White AF subjects. CHA_2_DS_2_-VASc = **C**ongestive heart failure, **H**ypertension, **A**ge ≥75, **D**iabetes, **S**troke/ transient ischemic attack, **V**ascular disease, **A**ge 65–74, **S**ex (female). **c. Geographic Variation of Differences (White–Black) in untreated rates among Medicare patients with non-valvular atrial fibrillation and CHA**_**2**_**DS**_**2**_**-VASc score ≥2 (N = 2,390,830).** Differences in untreated rates between Black and White AF subjects in each US state. CHA_2_DS_2_-VASc = **C**ongestive heart failure, **H**ypertension, **A**ge ≥75, **D**iabetes, **S**troke/ transient ischemic attack, **V**ascular disease, **A**ge 65–74, **S**ex (female). **d. Geographic variation of statistically significant differences (White- Black) in untreated rates among Medicare patients with non-valvular atrial fibrillation and CHA**_**2**_**DS**_**2**_-VASc score ≥2 (N = 2,390,830)*. CHA_2_DS_2_-VASc = **C**ongestive heart failure, **H**ypertension, **A**ge ≥75, **D**iabetes, **S**troke/ transient ischemic attack, **V**ascular disease, **A**ge 65–74, **S**ex (female) *The majority (93%) of counties that have significant differences are those where more Black patients are untreated than White patients and the difference is 0%-20%.

Eligible Black patients were more likely to be untreated with OACs (56.1%; hereafter using untreated in general) compared to White patients (47.4%, p-value<0.0001; **Table 2**). Among the treated patients, Black patients were more likely to be treated with warfarin (Black vs. White patients: 68.2% vs. 63.9%, p-value <0.0001) while White patients were more likely to treated with DOACs (Black vs. White patients: 31.8% vs. 36.1%, p-value<0.0001; **Table 2**). Untreated rates were the highest in the southeast US among Black patients (**Fig 2A**). Untreated rates varied by county among White patients with higher untreated rates in counties with sparse population (**Fig 2B**). Black patients generally had higher rates of non-treatment with OACs compared to White patients, irrespective of region (**Fig 2C**).

**Table 2:** Oral anticoagulant treatment patterns.

	Black Patients		White Patients		P-Value
	N	%	N	%	
**All Patients**	140,502	100%	2,250,328	100%	
Untreated	78,877	56.1%	1,066,395	47.4%	<0.0001
Treated	61,625	43.9%	1,183,933	52.6%	<0.0001
OAC Warfarin	42,007	68.2%	755,975	63.9%	<0.0001
DOAC	19,618	31.8%	427,958	36.1%	<0.0001

OAC = oral anticoagulant; DOAC = direct oral anticoagulant

Most differences in treatment rates at the county level were not statistically significant (**Fig 2D**). The few counties with significant differences were dispersed throughout the US and are not concentrated in a single state, though most correspond to highly populated areas such as Las Vegas, Los Angeles, New York City, and Miami (**Fig 2D**).

**Fig 3:**
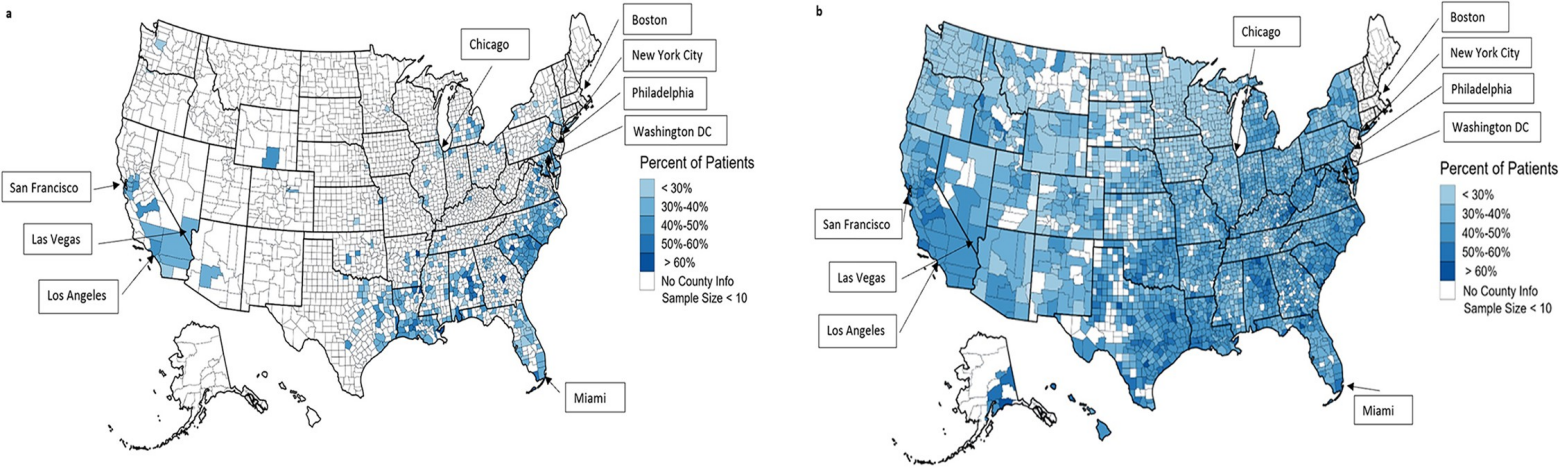
**a. Percentage of black patients using a DOAC medication with non-valvular atrial fibrillation and CHA_2_DS_2_-VASc score ≥2 (N = 61,625).** Percentage of black patients using DOAC medication. CHA_2_DS_2_-VASc = **C**ongestive heart failure, **H**ypertension, **A**ge ≥75, **D**iabetes, **S**troke/ transient ischemic attack, **V**ascular disease, **A**ge 65–74, **S**ex (female). **b. Percentage of white patients using DOAC medication with non-valvular atrial fibrillation and CHA**_**2**_**DS**_**2**_**-VASc score ≥2 (N = 1,183,933).** Percentage of white patients using DOAC medication. CHA_2_DS_2_-VASc = **C**ongestive heart failure, **H**ypertension, **A**ge ≥75, **D**iabetes, **S**troke/ transient ischemic attack, **V**ascular disease, **A**ge 65–74, **S**ex (female).

Among those treated with OACs, warfarin was the predominant treatment for both Black (68.2%) and White (63.9%) patients (**Table 2**). In the southeast, DOAC treatment was lower among Black patients compared to White patients (**Fig 3A and 3B**).

Variations within each state highlight areas with the highest unmet need. For example, areas of Michigan had higher DOAC treatment rates among Black patients in contrast to other areas of Michigan with low DOAC treatment rates among Black patients (**Fig 3A**). Similarly, there was low overall DOAC use in western North Carolina, but higher use in central and east North Carolina especially among White patients (**Fig 3B**).

## Discussion

We sought to determine whether previously observed racial differences in OAC treatment for AF varied at the state or county level among patients with drug coverage in the US. Our study has several notable findings. First, we found that differences in OAC treatment rates between races were highly heterogeneous across and within states. Although Black patients (vs White patients) were sicker (higher CCI, HAS-BLED and CHA_2_DS_2_-VASC scores) and younger, Black patients had higher OAC untreated rates. In addition, Black patients were more likely to be treated with warfarin while White patients were more likely to be treated with DOACs. Lastly, the higher untreated rates of OAC use among Black patients, particularly in states that comprise the “stroke belt” of the US, which in part, maybe a factor of increased strokes in a region of the country where Black patients predominantly live.

Prior studies have evaluated the utilization of OACs including DOACs in patients with AF as function of race and ethncity. In the Outcomes Registry for Better Informed Treatment of AF II (ORBIT-AF II), Black patients with AF, after adjusting for clinical characteristics, were significantly less likely to receive DOACs (adjusted odds ratio, 0.63 [95%CI,0.49–0.83) compared to White patients; similar findings were demonstrated when adjusting for socioeconomic factors [27]. In the veterans health administration, Essien et al. demonstrated that underrepresented racial and ethnic groups including Black patients with AF were less likely to initiate any OAC for stroke reduction (adjsuted odds ratio, 0.90; 95%CI 0.85–0.95) and were less likely to initiate DOACs (adjusted odds ratio, 0.74; 95%CI 0.69–0.80) compared to White patients [28]. In the Get With Guidelines AF Registry, Black patients with AF were less likely to receive any oral anticoagulant (adjusted odds ratio, 0.75; 95% CI, 0.68–0.84), including the direct oral anticoagulants (adjusted odds ratio, 0.73; 95% CI, 0.65–0.82) compared to White patients [29].

Additional studies investigating geographic variations in OAC utilization have documented large variations in OAC by region in the US. Hernandez et. al. documented the highest probability of initiating OAC in the Northeast US [mean adjusted probability (MAP)-0.54)] and Midwest US (MAP-0.54) compared to the lowest probability in the southeast US (MAP- 0.47) [15]. In addition, Hernandez et. al documented that the highest probability of being prescribed a DOAC was in the Southeast US (MAP- 0.50) and lowest in the Midwest (MAP-0.36). Atwater et al., using 3-digit zip codes, demonstrated the highest percentage of untreated patients with non valvular AF and a CHA_2_DS_2_-VASC ≥2 was in the Southern region of the US [20]. Claxton et al. demonstrated in the Optum Clininformatics datamart database (which included race) that the age-, sex-, and race-standardized incident stroke rates where highest (5.0/1000 person years) in East North Central United States (Indiana, Illinois, Michigan, Ohio, and Wisconsin) from the years of 2009–2015 and not the East South Central US (4.5/1000 person years) and South Atlantic US (4.1/1000 person years) [30]. Claxton and colleagues also demonstrated that patients in the East South Central and South Atlantic US, which typically comprise the classic “stroke belt”, were 14% and 10% less likely to be prescribed oral anticoagulation; these results were not broken down as a function of race and ethnicity.

Our study adds to prior work [20] on geographic variation by providing observed differences in untreated rates between White and Black patients. We demonstrate geographic variation in frequency of OAC treatment among White patients, and the higher undertreatment in the southeastern US that is primarily driven by undertreatment of Black patients. Furthermore, we provide county-level data which demonstrates several areas, primarily clustered in large dense populated areas throughout the US and within different areas of the southeastern US, where the differences in untreated rates between Black vs White patients with AF were significant. Differences in OAC treatment by race and geographic region are heterogenous across counties within states, particularly within the southeastern US.

The additional level of granularity highlights areas of unmet need within each state and opportunities for potential quality improvement initiatives. Quality improvement initiatives aimed at improving OAC utilization not only as a function of race, but also local geographic region could lead to a reduction in the burden of AF related ischemic strokes and their associated morbidity, mortality, and economic impact. The opportunity to develop customized interventions aimed at targeting the origins of decreased utilization of OAC in patients with AF as a function of race and geography is possible, but only with a better understanding of the determinants that continue to drive racial and geographic differences in OAC utilization. Research geared at understanding specific barriers and preferences for OAC use are needed from multiple perspectives including patients, clinicians, health systems, as well as better understanding of how structural racial inequalities continues to drive social determinants of health in specific geographic regions, which may, in part, play a role in the underutilization of oral stroke reduction therapies in underrepresented racial and ethnic groups with AF.

Lastly, the implications of this study’s findings and in the context of the prior work by Claxton et al. suggest that additional work is needed to better understand the contemporary stroke risk attributable to AF in the US as a function of race, ethnicity, and geographic region. Furthermore, an improved understanding of contemporary OAC utilization patterns among different racial, ethnic, and geographical regions is needed in an era preferential use of DOACs for stroke reduction therapies in patients with AF.

## Limitations

While claims data are valuable for examination of healthcare outcomes, they are collected for billing and not research purposes. Lack of certain clinical, laboratory, and disease-specific parameters, patient out-of-pocket costs; and samples or over-the-counter medications may lead to potential under-reporting. The study only included data until 2017 as this was the most recent data at the time of the initial study. Change of treatment landscape in recent years was not captured. Patients were assigned to a treatment cohort (warfarin or DOAC) based on the first prescription received; therefore, the results reflect initial treatment, not final OAC treatment. This study assessed data for a US Medicare population >65 years of age with prescription drug coverage; the results may not apply to younger patients, those with other forms of insurance, and/or those without drug coverage. Since the initiation of this study, clinical guidelines for OAC treatment for NVAF have changed. Majority of the study patients were white patients; black patients was only around 6%. Treatment for patients with AF and a CHA_2_DS_2_-VASc score ≥2 (which matched guidelines during the study period) were reported. Yet current standards now recommend OAC treatment for men with a CHA_2_DS_2_-VASc score ≥2 and women with a CHA_2_DS_2_-VASc score ≥3. Due to the updated recommendations and trends in OAC utilization, current use of DOACs is possibly more predominant than that observed in our study [23, 24, 31]. Geographic variation may have been influenced by other factors not investigated here, such as socio-demographic characteristics, hospital characteristics, and patient access to resources. Lastly, it was difficult to capture any patient relocations during the study period, therefore we cannot examine the impact on the results.

## Conclusion

Our study highlights differences in OAC utilization by county in the US and by race. The observed differences point to areas of unmet need for both Black and White patients. Future studies are needed to understand determinants of OAC use, overall and individually, particularly among Black patients with AF at the local, regional, and national levels.
